# Supplementary material for: Are carbon emissions trading and green financial instruments synergistic? -Comprehensive quantitative research based on content analysis
Source: PLoS One. 2024 Mar 7;19(3):e0298601. doi: 10.1371/journal.pone.0298601 (PMC10919601; doi:10.1371/journal.pone.0298601)
Supplement: S1 Data — (ZIP) [file pone.0298601.s001.zip › Data/地级市Z_F工作报告.zip!/║■─╧/╘└╤⌠╩╨/└┤╫╘╣1⁄2╓┌║┼í╛┬φ┐╦╩2╛▌═°í┐_2011.docx]

各位代表：

我代表市人民政府，向大会作政府工作报告，请各位代表连同《岳阳市国民经济和社会发展第十二个五年规划纲要》（草案）一并审议，并请各位政协委员和其他列席人员提出意见。

一、“十一五”国民经济和社会发展回顾

在中共岳阳市委的坚强领导下，全市人民深入贯彻落实科学发展观，认真执行省委、省政府工作部署，努力践行民本岳阳执政和发展理念，团结奋斗，开拓创新，各个方面取得重大成就，超额完成了“十一五”发展目标任务。

经济实力实现历史性跨越。全市GDP跨上千亿台阶，达到1491亿元（预计数，下同），年均增长14.1%。财政总收入跨上百亿台阶，达到139.6亿元，年均增长27.5%。社会消费品零售总额达到513亿元，年均增长19%。累计完成全社会固定资产投资2400亿元，年均增长34.1%。规模工业增加值达到740亿元，年均增长19.4%。农业生产连年丰收，粮食总产稳定在320万吨左右，水产品总产量保持全省第一。县域经济快速发展，占全市GDP比重提高到61%。金融机构存贷款余额分别达到785亿元和437.2亿元。我市被国家统计局评为新中国成立60年60个城市发展代表。

结构调整取得实质性进展。2010年与2005年相比，二产业对经济增长贡献率提高10.2个百分点，达到69.1%。财政总收入占GDP比重提高2.9个百分点，达到9.4%。税收占财政总收入比重提高9.6个百分点，达到84.2%。地方工业增加值占全部工业增加值比重提高15个百分点，达到76.6%。园区工业增加值占全市规模工业增加值比重提高31.6个百分点，达到43.9%。非公经济占GDP比重提高15.3个百分点，达到57.5%。城镇化率提高8.1个百分点，达到48.1%。全市万元GDP能耗累计下降21%，主要污染物总量累计减排6%。三次产业结构由20:46.4:33.6调整为13.3:53.6:33.1。

基础设施得到全方位改善。城镇建成区面积新增267平方公里，达到410.2平方公里。中心城区新建改建15条主干道路，亮化美化200多条小街巷，建成文化艺术会展中心等一批标志性工程。续建洞庭湖风光带、环南湖旅游走廊。体育中心开工建设。建成19个新农村示范片、292个示范村。完成各类水利工程25.6万处，加固堤防310公里，整治大小水库530座。新建改建农村公路11213公里，所有乡镇和88.6%的行政村通水泥路。武广客运专线岳阳段及岳阳东站建成运营，荆岳长江大桥、汨罗江大桥和柳林江大桥竣工通车，随岳、岳长、岳常、通平、石华、临岳6条高速公路相继开工。建成投产500千伏变电站1座、220千伏变电站3座。通讯、燃气等基础设施大为改善。

改革开放迈出坚实性步伐。市属国有企业改制基本完成，5年累计完成94家。成品油税费改革全面完成。城区财税体制进一步理顺。市县政府机构改革、省管县财政体制改革、文化体制改革、集体林权制度改革和医药卫生体制改革扎实推进。新引进国内外500强企业20家；城陵矶口岸开通海轮航道和“五定”班轮航线，城陵矶港成为海港，对台直航达成协议；成功举办奥运火炬传递、第二届湘商大会、湖南旅游节开幕式、“五湖牵手五岳”旅游同盟大会、君山十大爱情故事评选颁奖晚会；加强与6个国际友好城市交往，岳阳开放形象进一步提升。

社会建设实现整体性推进。城镇居民人均可支配收入、农民人均纯收入分别达到17248元和5926元，年均分别增长9.8%和10.7%。“两基”人口覆盖率100%，高中阶段教育进入普及期，职业教育跃居全省第一，高等教育发展步伐加快。组建工程技术研究中心、产学研战略联盟等创新平台42个，实施自主创新引导和产学研专项166个，《石脑油催化重整成套技术开发与应用》荣获国家科技进步一等奖。新型农村合作医疗参合率98.4%，5年补偿7.4亿元。改扩建175家乡镇卫生院，新建村卫生室133个、社区卫生服务中心28家、社区卫生服务站56个。21.3万农村老人开始领取养老金。1.5万五保户集中供养。帮助1.1万户农村困难群众新建改建住房。58万人参加志愿服务行动，慈善募捐8900万元，建立爱心超市11家。投入4300万元，改善123个社区办公服务用房。精神文明建设不断加强，公共文化服务体系建设加快，群众性文化活动蓬勃发展，《今天有客来》和《春雨》荣获“群星奖”金奖，汨罗江畔端午习俗进入世界级非物质文化遗产名录。全民体育健身运动深入开展，全省第十一届运动会岳阳总分排名比上届前进6个名次。人口计生工作保持全省先进，计划生育手术并发症患者扶助制度在全国率先试点。国防动员和民兵预备役工作得到加强。统计、民族、宗教、对台、史志、档案、保密、气象、人防、烟草、邮电、盐业、老龄、残联等各项工作取得新成绩。

过去5年，我市为全面建成小康社会又迈出坚实一步，必将激励我们在科学发展与民本岳阳和谐崛起的道路上继续奋勇前进。

回顾5年来的工作，我们特别突出了以下几个重点：

（一）突出科学谋划。按照市委确定的民本岳阳“六大目标”和“九项工程”，从大处着手谋划岳阳大局发展。一是在国家战略中谋划岳阳发展平台。抓住国家批准长株潭“两型社会”综合改革试验区的机遇，成功争取678.5平方公里纳入核心区规划，建立湖南城陵矶临港产业新区；岳阳经济技术开发区晋升国家级；汨罗循环经济工业园被列为全国首批“城市矿产” 示范基地；云溪工业园成为国家新型工业化产业示范基地；屈原管理区进入全国现代农业示范区；国家级台湾农民创业园落户岳阳县。我市被国家定为加工贸易梯度转移重点承接地、商标战略实施示范城市、知识产权试点城市、磁力设备质量监督检验中心。争取到这些牌子，就争取了国家一系列的政策支持。二是在发挥岳阳优势上谋划大项目大发展。发挥中央省属骨干企业优势，争取了炼化一体化、华能三期、岳纸40万吨等重大项目建设；发挥民营企业机制活的优势，积极支持与资本市场对接，5年新增上市企业6家，3家企业在天津股权交易所挂牌交易；发挥通江达海优势，引进战略投资者建成城陵矶新港，启动长江航道疏浚，形成大通关网络；发挥文化优势，建成岳阳楼新景区，争取了国家和省里支持建设屈子文化园。三是在科学规划、优化布局上谋划长远发展。在完成城市规划及土地利用总体规划修编的基础上，推进产业规划、城市规划与土地利用规划“三规合一”。完成中心城区155平方公里控详规划编制，实现了城市控详规划全覆盖。中心城区按照“东拓、西连、南延、北靠、中提”，调整产业布局，岳阳楼景区周边工业逐步实施“退二进三”，南湖风景区逐步退出工业、调整为以休闲旅游为主，发展北边沿江工业走廊。坚持“一核三圈”城镇梯级发展，初步形成了以中心城区为核心、县城和中心镇为节点、各乡镇协调发展的新型城镇体系。

（二）突出项目建设。认真落实市委“月月上项目、年年创大业”的要求，把项目建设作为经济增长的第一推动力。一是大力开展招商引资。5年引进内资项目1452个，到位资金615.7亿元；利用外资4.7亿美元。二是全力争取国家投资。5年争取国家和省里资金356.9亿元。荆岳铁路即将开工建设，小墨山核电项目列入国家核电中长期调整规划。三是努力激活民间投资。拓宽领域，非禁即入，鼓励全民创业。5年民间投资1440亿元，年均增长44.6%。四是着力优化项目服务。连续开展“项目建设与项目服务年” 和“机关效能建设年”活动，深入开展联手帮扶企业和产业发展升级行动，认真落实项目建设“五个工作环节”和“六个一”服务，努力营造规范、高效、透明的发展环境。五是用项目建设检验工作成效。每年召开项目建设流动现场会，看典型、比成效、找差距；重奖招商引资先进单位和个人，激励各级各部门争投资、上项目。5年实施投资过5000万元的重大项目1161个，多年为之努力的一批重大项目得到落实，不仅实现了当前保增长，而且增强了发展后劲。

（三）突出生态建设。着力打造更具竞争力的生态环境，促进可持续发展。一是按照“两型社会”建设的要求，保护自然生态环境。发挥在岳的全国和省人大代表、政协委员作用，大力呼吁洞庭湖综合治理，争取项目489个、投资48.1亿元。加强环境污染治理，全面完成洞庭湖周边102家造纸企业整治任务和57个湘江流域综合整治项目，建成10个污水处理厂，淘汰落后产能企业34家，关停“十五小”污染企业97家，启动环南湖截污管网建设，全面完成“十一五”节能减排任务。大力开展植树造林，增加森林碳汇，全市森林覆盖率达到45.3%。综合整治农村土地1.6万公顷。君山区被授予“中国野生荷花之乡”。二是按照市场经济发展的要求，优化金融生态环境。2007年启动金融生态区建设，打击恶意逃废债务和非法集资行为，积极开展金融诉讼案件集中执行活动，我市被评为 “中国金融生态城市”。深化银企合作，建立完善7大投融资平台，创办平江汇丰、华容鸿基村镇银行等5家新型金融机构。全市保险公司发展到32家。三是按照和谐社会建设的要求，提升人文生态环境。大张旗鼓开展以创建全国文明城市为龙头的“五创提质”行动，城区更加整洁、有序、秀美。大力加强社会治安综合治理，深入开展“扫黑除恶”、禁毒禁赌、扫黄打非和打击传销等专项行动，治安民调年年进位，2010年综治考评全省排名第一。开展大接访，有效解决了一批热点难点和历史遗留问题。建立健全应急预防体系，切实抓好安全生产，社会大局保持和谐稳定。

（四）突出改善民生。注重解决群众最现实、最关心、最直接的利益问题。一是认真落实各项惠民政策。克服困难，抓好配套，支农补贴及时发放；企业军转干部生活补助进一步提标；企业退休人员养老金连续5年提高待遇，人均月增加570元；城区低保标准由2005年的每月180元提高到260元，建立农村低保制度，月标准达到110元。二是坚持不懈为民办实事。全面完成省、市实事任务。新增城镇就业25.4万人。解决100.5万农村人口饮水安全问题。新建改建和筹建廉租房、经济适用房165万平方米。洞庭湖区捕捞渔民全部上岸定居。移民后期扶持23万人，5年投入9亿多元。新建4319个广播电视村村通工程，每年免费送戏下乡900多场、电影下乡3.5万多场。三是创造条件让发展成果更多惠及民生。在全国率先实现零就业家庭动态清零，率先推行社区卫生“服务零距离、药品零利润、医患零纠纷”，在全省率先建立大病医疗救助基金，率先建立医患纠纷第三方调处机制，率先实现乡乡有敬老院目标，率先在农村实施大面积危房改造。这5年，全市财政民生支出268.6亿元，占财政总支出的61.3%，惠及全民的民生保障体系初步形成，人民群众得到更多实惠。

（五）突出管理创新。按照依法行政、转变政府职能的要求，努力做到管理精细化，更好地服务人民群众、服务发展。一是按照“规范有序”的原则，建立健全各项制度，规范权力运行。自觉接受人大和政协监督，认真办理人大议案、代表建议和政协建议案、委员提案。认真落实《湖南省行政程序规定》，出台市政府重大行政决策规定，推进科学民主决策。健全完善市政府投资项目管理、财政性资金管理、土地管理、规划管理等系列意见，加强招标投标监督，全面启动规范权力运行制度建设。减少行政审批项目622项。市直232个社团基本实现“五脱钩”。加强和改进政府绩效评估，认真执行“有错无为问责”，不换状态就换人，5年行政处分1192人，其中开除公职173人。实行重大项目审计、监察全程跟踪等制度，深入推进廉政建设和反腐败工作，认真开展政风行风民主评议，公务员队伍建设取得新成效。二是按照“便民利民”原则，创新服务平台，提高办事效率。建立完善政务服务中心，搭建信息公开、便民服务、市场交易、电子政务“四大”平台。在全省率先建立部门行政许可职能集中行使和建设工程项目联合审批、联合验收制度，全面推行投资项目审批委托代办和一票制收费，所有行政审批项目办理时限压缩30%。三是按照“竞争择优”原则，设立内部模拟市场，公共事务逐步实现从养人到养事转变。加强公共财政体系建设，在预算编制中对有定额可依的环卫、绿化、路灯、市政等单位，根据实际工作量，定额编制预算，不再另行安排人员支出；岳阳楼新景区管理、城区新建改建道路等，实行项目管理，购买公共服务，发挥财政资金的最大效益。

各位代表，“十一五”时期，我市经济和社会发展的重大成就，是克服重重困难取得的，来之不易。我们有效抵御了国际金融危机的冲击，战胜了低温雨雪冰冻灾害和黄盖湖、汨罗江、湘阴湘江特大洪涝灾害，积极应对错综复杂的社会矛盾和问题。这些成就的取得，是中共岳阳市委坚强领导、全市上下团结奋斗的结果。在此，我代表市人民政府，向全市广大工人、农民、干部和知识分子，向各民主党派、工商联、无党派人士、各人民团体和社会各界人士，向驻岳人民解放军、武警、消防部队指战员和政法干警，向所有关心、支持和参与岳阳改革建设的人们，表示崇高敬意和衷心感谢！

在充分肯定成绩的同时，我们也清醒地认识到，我市经济社会发展还存在不少问题。主要是：产业结构性矛盾仍然比较突出，三产业比重偏低，新兴产业比重偏小；农业基础设施比较薄弱，农村公共事业亟待加强；节能减排、安全生产压力加大；保障和改善民生任务繁重，群众在上学、就医、住房、养老等方面还有许多实际困难；政府职能转变与科学发展的要求相比还有差距，行政效能建设、机关作风建设和廉政建设还需进一步加强。对此，我们一定高度重视，进一步采取措施，切实加以解决。

二、“十二五”面临的机遇、挑战与对策

“十二五”时期，是全面建设小康社会的关键时期，是加快转变经济发展方式的攻坚时期，也是岳阳在新的起点上进一步开创科学发展、富民强市新局面的重要机遇期：一是中央明确提出加强改革顶层设计，着眼于解决体制机制根本性问题，有利于我们突破重点领域改革。二是国家加快转变经济发展方式，更加注重生态环保，有利于我们争取核电等新兴能源项目，加快航运物流业、旅游业和循环经济发展。三是国家加强农田水利建设，加大粮食主产区投入和利益补偿，有利于我们争取更多项目、更多投入提高农业综合生产能力，加快传统农业向现代农业转变。四是国家着力改善民生、扩大内需，有利于我们发展第三产业，扩大城乡就业。五是国家促进中部崛起力度加大，有利于我们争取更多的政策支持。六是湖南全面推进“四化两型”战略，有利于我们发挥比较优势，加快承接沿海产业转移、对接长株潭。七是岳阳发展后劲大为增强，特别是全市上下有加快发展的强烈愿望和坚定信心，有利于转化为发展的强大动力。

与此同时，我们也面临严峻挑战：一是国内外经济发展环境存在不可预见的因素，必须审时度势，增强工作的前瞻性，积极应对。二是改革发展的压力越来越大，改善民生的要求越来越高，必须不断解放思想，用发展的办法解决前进中的问题。三是转方式、调结构任重道远，必须在新的起点上谋划新一轮发展。四是土地、资金、能源等要素趋紧，必须突破瓶颈，吸纳有效资源配置到最需要的地方。五是国家对节能减排、保护环境的要求越来越严，必须发展绿色经济，倡导低碳消费。六是岳阳地处长株潭城市群和武汉城市圈中间，不在发展中崛起，就会成为发展的“过水之丘”，必须主动对接，抢占发展先机。七是社会组织管理和维护稳定的任务艰巨，必须创新社会管理，促进经济社会协调发展。

面对机遇和挑战，我们既要增强机遇意识，又要强化忧患意识，始终扭住经济建设这个中心，不放松、不动摇、不折腾。坚持以科学发展、富民强市为主题，以加快转变经济发展方式为主线，按照省委、省政府的战略部署，大力推进“四化两型”和“五市一极”建设，努力实现岳阳经济转型升级、更大更强，把岳阳建设成为现代工业大市、现代农业强市、现代航运物流旺市、现代旅游热市、现代生态宜居城市和湖南经济新增长极，为全面建成小康社会打下具有决定性意义的基础，让全市人民生活得更加幸福、更加美好！

三、“十二五”的主要目标与任务

主要奋斗目标是：

经济实力更强。力争实现三个翻番：经济总量翻番，达到3000亿元；财政收入翻番，达到300亿元；城乡居民人均收入翻番，分别达到34496元和11852元。

发展方式更优。力争实现三个提高：提高二三产业占GDP的比重，一二三产业结构调整为9:55:36；提高高新技术产业增加值占GDP的比重，超过25%；提高财政收入占GDP的比重，超过10%。

生态环境更美。努力做到三个减少: 万元GDP能耗累计减少15.4%；主要污染物排放累计减少1.6%；万元GDP二氧化碳排放量累计减少10%，空气质量优良率达到90%以上。

文明程度更高。政治更民主、政务更透明、队伍更纯洁、制度更科学、风气更清新、社会更和谐，力争成功创建全国文明城市。

人民生活更好。城乡居民收入水平不断提高、享受到的公共服务不断增加、生活环境不断改善，增强全市人民的幸福感和自豪感。

主要任务是：

（一）建设现代工业大市

以项目建设、技术改造、管理创新为抓手，加快推进新型工业化。力争到2015年全市工业主营业务收入突破6000亿元，规模工业增加值1500亿元，工业对GDP贡献率50%以上。大力推进“千百十”工程，形成石油化工、食品加工两个产值过千亿元产业，10个产值超百亿元园区，70个以上主营业务过10亿元企业。

加快提升传统产业。积极发展符合国家产业政策、符合国家节能减排要求，有市场、有规模、有效益的传统产业。支持企业加大技术改造、加快管理创新、加强产业联合；鼓励企业提高品牌化、“两型化”、信息化水平，增强传统产业的竞争力和对区域经济的支撑力。把石化产业作为岳阳最重要的产业，围绕炼化一体化，加速发展特色化工产业链。到2015年，原油加工能力达到1500万吨、争取2000万吨，石化总产值1800亿元、争取2000亿元。大力发展食品加工业，到2015年，总产值1200亿元以上。同时，着力提高机械制造、造纸印刷、纺织服饰、建筑材料的加工深度和附加值，推动产业升级。

大力发展新兴产业。紧紧抓住国家“十二五”支持战略性新兴产业发展的大好机遇，加大对新兴产业的招商力度和财政支持力度，积极实施商标战略、专利战略和科技创新工程。大力发展新能源、新材料和文化创意产业。力争到2015年再生资源总产值480亿元以上、电子信息产业总产值380亿元以上、生物医药产业总产值200亿元以上、先进装备制造业总产值150亿元以上，成为全市的优势产业。

鼓励发展中小企业。在加快龙头企业发展的同时，促进专、精、特、新中小企业快速发展，支持企业提档升级。促进中小企业与大型企业协作配套、共同发展，努力形成“树大成林、干强叶茂”的工业生态。以此促进产业集群发展，推动全民创业，扩大社会就业。

聚集发展园区工业。进一步明确各地园区产业定位，推进项目入园、产业集聚，做长产业链。支持上市公司、优势企业、特色产业创办工业园。发挥国家级品牌优势，大力推进岳阳经济技术开发区、汨罗循环经济工业园、云溪精细化工园发展。发挥城陵矶临港产业新区通江达海、先行先试优势，建设现代新港、产业新区、滨江新城。形成华容、临湘等10个过百亿的工业园区。

（二）建设现代农业强市

以农民增收为核心，以统筹城乡发展为主线，加强现代农业和新农村建设。确保粮食安全，抓好“菜篮子”工程，提高产业化水平。到2015年，农业增加值270亿元，年销售收入过10亿元的龙头企业20家以上，形成食品加工、棉花加工、竹木加工、作物种子和农机加工等产业链。涉农上市公司达到8家以上。打响君山银针等岳阳地域品牌。

大力推进现代农业建设。积极推进标准型、规模型、加工型、品牌型、安全型和休闲型农业，促进农业发展方式转变。提高机械化作业、专业化服务水平。培育壮大龙头企业。抓好农超对接，让岳阳农产品更多地进入超市。推进农村土地有序流转。实施新增粮食产能工程、环洞庭湖基本农田建设重大工程等项目。推进屈原国家级现代农业示范区、湘阴全国农业标准化示范县、君山全国农产品加工示范基地、岳阳县台湾农民创业园建设。

大力推进新农村建设。强化乡村规划，推进乡村居民点集中布局，逐步形成有特色的地域建筑风格。加强农村公路、水利、沼气、电网、互联网、广播电视等基础设施建设和管理，改造农村危房。大力改善农村卫生环境，推进农村“四清”、“四改”。加快发展农村文化、教育和卫生事业，抓好农民技能培训，提高农民素质。

大力推进城乡统筹发展。加大财政对“三农”的投入，整合涉农资金，确保安全有效使用。深入推进农村贫困人口“两项制度”衔接工作。深化农村信用社改革，积极发展村镇银行和资金互助社，健全农业保险制度。深入开展“万企联村、共同发展”活动。探索农村社区建设，逐步推进农村居住社区化。促进城乡规划、产业发展、基础设施、环境保护、公共服务有效对接，推动城市资本、技术优势和农村资源优势相结合，形成城乡互动互补与一体化发展格局。

大力推进县域经济发展。进一步落实促进县域经济发展的各项措施，发展特色经济、非公有制经济、开放型经济和劳务经济。支持办好县域工业园，坚持“工业入园、园区兴县”。推进县城和中心镇扩容提质，建设一批经济强镇和特色小镇，争取更多县（市）区进入全省10强、20强。

（三）建设现代航运物流旺市

充分发挥区位优势和水运优势，把岳阳建成长江中游区域性航运物流中心。力争到2015年全市集装箱达到40万标箱，货物吞吐量1.5亿吨；现代物流业增加值突破300亿元，占GDP比重10%以上；全社会物流成本占GDP比重比“十一五”末下降2个百分点。

建设大口岸。推进沿江、沿湖港口群建设，新建城陵矶新港二期工程，改扩建城陵矶老港区。完成洞庭湖水上应急救援指挥中心二三期工程。加快国家级出口加工区报批与建设，开辟口岸新航线，推进对台直航常态化。加快建设公路口岸。规划建设航空口岸。

培育大市场。认真实施商业网点规划，完善市场体系。建设和提质一批专业市场，繁荣特色商贸街区，改造集贸市场。支持临湘、华容、平江建设省际边贸物流园和专业市场。依托大市场建设，集散物流、繁荣物流。

形成大流通。打通进出通道，完善物流交通体系。培育引进物流大企业，吸引国内外优势物流企业建立基地和营运中心，带动中小物流企业发展。建设一批集仓储、配送、电子商务、商品加工为一体的现代物流基地。积极发展第三方物流。建立物流电子交易平台，加大与境内外的商务合作。推进物流业与制造业联动，延伸物流产业链。

（四）建设现代旅游热市

立足旅游资源和品牌优势，优化产品结构，完善服务体系，加大营销力度，加快项目建设，大力发展休闲旅游，促进岳阳旅游由过境游向目的地游转变，努力把旅游业培育成支柱产业。力争5年旅游产业项目投资160亿元，到2015年旅游总收入300亿元以上，占GDP比重10%以上。

开发旅游产品。提升环湖旅游，以“楼岛湖”为主体，联合申报洞庭湖国家级旅游度假区，形成主打旅游品牌。拓展沿江旅游，开发屈子文化园、顺天洋沙湖度假村、盘石洲度假区、左宗棠故居、青山岛等旅游资源，建设对接长沙的休闲旅游带。开发名山民俗旅游，整合五尖山、大云山、幕阜山、福寿山等国家级、省级森林公园生态旅游资源，挖掘巴陵戏、长乐故事会、九龙舞等地方特色文化，促进旅游与文化融合。推进城市与产业旅游，把城市作为大旅游产品来打造，开发工业旅游、特色种养基地、花卉苗木、地标建筑、桥梁景观等旅游产品。

提升旅游服务。完善旅游交通，加快高速公路、主要交通干线与重点旅游景区对接。做特湖鲜美食，建设五星级宾馆，鼓励发展家庭旅馆和汽车旅馆，规范发展影视、歌厅、酒吧、茶艺、咖啡馆等休闲娱乐业，打造高水平、有特色的地方综艺节目和商业服务中心。开发特色旅游商品，拉动旅游购物消费。

创新旅游营销。重视旅游营销策划，把景区变产品、产品变产业，把促销旅游产品与宣传城市紧密结合，扩大岳阳知名度。用市场手段办好汨罗江国际龙舟节、君山爱情岛旅游文化节等一批有地方特色的旅游节会。引进国内外知名旅游企业，开发旅游精品线路，对接热点旅游市场，在更大范围参与合作和竞争。

（五）建设现代生态宜居城市

按照集约高效、功能完善、环境友好的原则，大力推进新型城镇化，构建新型城镇体系，提高城镇建设和管理水平，把岳阳建设成为现代生态宜居城市。到2015年，全市城镇人口达到315万，城镇化率55%。

促进生态良好。抓好城区水系保护和治理，推进流域环境综合整治，加强洞庭湖湿地保护，建设“水美型”城市。新建一批城市公园，大力开展植树造林，积极实施沿湖、沿江、沿河“绿化生态”工程。加快开发君山生态资源，推进“跨湖”发展。严格实施节能减排，减少大气污染，让老百姓呼吸新鲜空气；加强城镇污水、生活垃圾、固体废弃物处理设施建设和运行管理，建立环境卫生保洁长效机制。

力求生活方便。抓好高速公路连接线和环城道路建设，拉通德胜北路等30多条主次干道，加快交通拥堵地段立交桥和地下停车场建设。完善城市管道、水电、通讯、环卫等基础设施，扩大城镇承载产业发展、吸纳就业的容量。加快旧城和小街小巷提质改造。配套完善医院、学校、商场、广场、人防、游园、文化、体育等公益设施，让市民生活更方便、更舒适。

构建社会和谐。加强安全生产，完善社会治安防控体系，增强市民安全感。创新城市管理，规范城市交通秩序和市场秩序。提升城市品位，培育特色城市文化，塑造城市个性与形象，增强市民现代文明意识。让岳阳更文明、更和谐、更秀美。

（六）扩大投资与消费

扩大有效投资，引导消费升级，集中抓一批影响全局、支撑长远发展的大项目，力争5年完成固定资产投资6500亿元；到2015年，社会消费品零售总额突破1000亿元。

扩大基础设施投入。交通：5年投资380亿元以上，加快构建“三纵三横”铁路网、“四纵两横”高速公路网、“八纵七横”干线公路网和便捷通达的农村公路网。建成石华、临岳等6条高速公路，争取开工建设平益高速。加强国省干线公路、农村公路建设和养护，完成芙蓉大道北拓至湘阴工程。建设荆岳铁路、城陵矶至松阳湖铁路专线，启动岳长、长平汨城际轨道和岳吉铁路建设，做好岳九、岳常铁路前期工作。加强长江和湘江航道岳阳段整治，提高通航等级。加快岳阳机场规划建设。水利：5年投资200亿元以上，加强防洪薄弱环节建设，抓好黄盖湖防洪抗灾工程。基本完成重点中小河流重要河段治理，全面完成中小型病险水库和规划内大中型病险水闸除险加固。实施大中型灌排泵站更新改造。搞好农村河道疏浚和小型水库清淤。切实加大铁山水库等城乡饮用水源保护力度，继续推进农村饮水安全工程建设。加快推进小型农田水利重点县建设。加强洞庭湖综合治理，推进城陵矶洞庭湖综合枢纽前期工作。能源：积极推进小墨山核电、华能岳阳二厂、福寿山和玉池抽水蓄能电站等前期工作，争取国家批准建设。力争启动一批太阳能、风力、沼气、生物质能发电项目。争取建设国家石油储备基地和湖南电煤储备基地。推进电网和成品油管道、输气管线建设。信息：积极支持 “三网融合”，大力发展物联网。加快信息化示范企业建设，促进信息化与工业化融合。提高交通、电网、环境监测等领域信息技术应用水平，大力推进社会管理、政务管理信息化，努力建设“数字岳阳”。

促进消费增长。提升消费能力，实施居民收入倍增计划，努力实现居民收入增长与经济发展同步、劳动效率与劳动报酬同步。优化消费环境，加强市场监管，依法打击和惩处制假售假、恶意炒作、哄抬物价等不法行为，切实保护消费者权益。发展新兴消费业态，培育文化、信息等消费热点，扩大汽车和住房消费。继续推进“家电下乡”和家电“以旧换新”，培育和扩大农村消费市场，努力把岳阳中心城区和湘阴县城建成一主一次的“两大”消费中心。

加强金融服务。强化社会信用体系建设，促进银企合作，扩大信贷规模，优化信贷结构，帮助银行做大做强。创新金融产品，扩大企业债券、中期票据、私募股权融资规模。坚决打击非法集资行为。加快引进银行和信托机构，大力培育地方中小金融机构。积极培育企业上市后备资源，利用资本市场直接融资，5年融资100亿元以上。加强保险创新，搞好保险理赔。

（七）加快社会建设和民生改善

顺应人民群众新期待、新要求，加快形成广覆盖、保基本、多层次、可持续的基本公共服务体系。

推进社会事业发展。一是发挥企业自主创新的主体作用，科技工作者重大专项研究和创新的主角作用，科技部门在服务、开放与合作中的主要作用，大力推进科技创新。二是加大教育投入，优化教育布局，加强师资队伍建设；重视发展学前教育，努力实现义务教育均衡发展，加快普及高中阶段教育，增强职业教育服务经济社会发展的能力，提升高等教育质量，关注特殊教育，加强社区教育，促进各类教育协调发展。三是深入实施人才强市工程，培养人才、吸引人才和留住人才，努力建设奋发进取的企业家团队、廉洁实干的公务员团队、积极作为的基层工作者团队，开创人才辈出新局面。四是加快构建覆盖城乡的公共文化服务体系，促进文化艺术产品创作和生产，建设市广电中心、图书馆、档案馆和青少年活动中心，加强互联网建设和管理，实现乡乡有综合文化站、村村有农家书屋。深化文化体制改革。五是积极开展全民健身运动，提高竞技体育水平，办好省第八届少数民族运动会，建好市体育中心。六是抓好人口计生工作，进入全省人口和计划生育“模范市州”。七是大力发展老龄事业和养老产业，建设养老服务中心，构建居家养老与机构养老相结合的养老体系，推进居家养老社会化、市场化。切实做好民族、宗教和外事、侨务工作。支持工会、共青团和妇联工作。

积极创新社会管理。一是健全基层民主政治制度，完善村民自治，加强社区建设，积极培育发展各类社会组织，支持社会组织参与公共服务和社会管理，加强和改善对新经济组织、新社会组织和虚拟社会管理。二是健全自然灾害、事故灾难、公共卫生事件、食品安全事件、社会安全事件的预防预警和应急处置体系。三是切实做好安全稳定和信访工作，完善人民调解、行政调解、司法调解联动的工作体系。四是加强社会治安综合治理，严密防范、依法打击各类违法犯罪行为，增强公共安全保障能力。五是加强精神文明建设，大力弘扬“先忧后乐、团结求索”的岳阳精神，坚定民本岳阳和谐崛起的价值追求，努力创建全国文明城市、国家环保模范城市、国家交通管理模范城市、国家绿化模范城市和国家社会治安综合治理先进市州。加强普法、国家安全和青少年思想道德教育。抓好国防动员和民兵预备役工作。

切实保障改善民生。一是扩大社会就业。坚持以项目扩大就业、创业带动就业、培训促进就业、政策扶持就业、服务保障就业。重点做好高校毕业生、城镇就业困难人员、退役军人就业工作。切实保障劳动者权益。二是完善社会保障体系。扩大各类保险覆盖面，突出解决农民工、被征地农民、城镇失业人员、城镇无业居民和残疾人等特殊人群的社会保障问题。建立健全城乡居民低保标准正常调整机制。全面普及新型农村社会养老保险。加强社会救济救助，积极发展以济困、助残、救孤为重点的社会福利事业。三是加强住房保障。加大保障性安居工程建设力度，加快棚户区和农村危房改造，大力发展公共租赁住房，缓解弱势群体的住房困难。四是做好卫生医疗工作。扎实推进医药卫生体制五项重点改革，优化配置卫生资源，建立健全城乡公共卫生服务、基本医疗服务、基本医疗保障和药品供应保障体系，普及社区卫生服务，积极防治血吸虫病，抓好重大传染病的防控，为群众提供满意的基本医疗卫生服务。让建设和发展的成果更多惠及民生。

（八）深化改革扩大开放

进一步解放思想、转变观念，努力实现改革开放新突破。

以更大决心深化改革。一是企业改革。全面完成市属国有企业改制任务。引进战略投资者参与公益性国有企业的改革重组，健全国有资产管理体制。引导民营企业加快股份制改造。二是投资体制改革。推行特许经营制、项目代建制、后评估制和责任追究制，增强政府投资的导向作用。三是财税体制改革。强化财政公共服务职能，实行全口径预算管理，建立财政偿债预算制度，调整和完善市对县特别是中心城区的体制。四是农村综合配套体制改革。继续推进乡镇机构、乡村财务管理、集体林权和国有林区林权制度改革。加快征地制度改革，保障失地农民合法权益。切实抓好乡村公益性债务化解。五是机关事业单位改革。进一步理顺职能、整合机构，着力构建权责一致、运转协调的大部门体制。积极推进事业单位分类改革。

在更大范围扩大开放。全面推进招商引资，主动对接国内外战略投资者，组织小分队招商、上门招商、以商招商、以亲招商，让更多的好项目、大项目落户岳阳。全面加强区域协作，鼓励有条件的企业到境外投资办厂、承包工程和劳务合作，努力提高经济外向度。全面打通进出口通道，畅通人流、物流、资金流和信息流，为扩大开放创造基础条件，增强各类生产要素集聚能力。全面培育开放素质，发展理念、制度设计、行为规范进一步与国际接轨，在更高层次扩大开放。

（九）加强政府自身建设

全面完成“十二五”各项目标任务，必须“忠诚于党和人民、忠诚于事业”，努力建设人民满意政府。

建设服务政府。加快转变政府职能，把主要精力放在经济调节、市场监管、社会管理和公共服务上。进一步精简和规范行政审批，认真解决审批职能交叉、权责脱节和多头审批等问题。深化政务公开，推进财政预算、公共资源交易、重大建设项目批准与实施、社会公益事业建设等领域的信息公开。建立完善电子政务系统，逐步实现网上办公、网上服务和网上监督。强化首问负责制、服务承诺制、限时办结制和全程代办制。增强为纳税人服务的意识。加强和改进社会管理，发挥群众组织和社会组织作用，促进社会和谐有序。

建设法治政府。坚持依法行政，加强规范权力运行制度建设，建立健全重大决策跟踪反馈和评估制度，严格按照法定程序、法定权限、法定职责行使权力。严格执行行政执法责任制和执法过错追究制，全面提高执法人员素质。凡事关民生、事关长远和事关全局的政府重大决策，及时向市人大报告、向市政协通报、向全社会公示，切实维护人大代表、政协委员、民主党派、工商联、无党派人士、人民团体和人民群众的知情权、监督权。

建设责任政府。按照“更高标准、更严管理、更具公信力”的要求，做到推动工作毫不放松，促进发展决不懈怠。建立科学合理的政府绩效评估指标体系和评估机制，推动机关效能建设科学化、制度化、长效化。严格落实责任追究制，激励政府工作人员恪尽职守、勤政为民，让政府各项工作经得起实践检验、人民检验和历史检验。

建设廉洁政府。加强对公务员的教育和管理，切实转变工作作风。严格执行党风廉政建设责任制，加强行政监察，坚决纠正损害群众利益的不正之风。继续深化工程建设领域突出问题和“小金库”、商业贿赂、公务用车、公务接待、公款出国境专项治理。强化对财政资金使用、国有资产运营、土地使用权出让、行政审批权运用等方面的监督和审计。降低行政成本，严格控制出国考察、培训，严格控制庆典、论坛、节会等方面的公共支出，以清廉形象取信于民。

四、扎实做好2011年工作，确保“十二五”良好开局

2011年，要为“十二五”开好局、起好步，形成岳阳经济转型升级、更大更强的强劲发展势头。全市经济社会发展预期目标是：地方生产总值增长11.5%，固定资产投资增长20%，社会消费品零售总额增长18%，财政总收入增长15%，城乡居民收入增长11.5%，人口自然增长率控制在6.5‰以内，各项社会事业同步发展，“五市一极”建设迈出实质性步伐。

在全面实施“十二五”规划的基础上，突出四个方面：

（一）会战“五区”。城区建设加力。城区是经济要素的重要承接地，推进城乡一体化首先是要增强城市的带动功能，加快中心城区及县城发展。一是拉骨架，拓展发展空间。城陵矶片：抓好港城连接道路两侧规划建设，推进港城融合。赶山片：启动“四纵五横”路网建设，推动城市南延。金凤桥片：新建金凤桥路等主干道，加快烟草物流园及配套路网建设。二是抓配套，完善城市功能。建成青年路跨王家河大桥，规划建设皇姑塘等立交桥。完成洞庭大道君山段提质改造。凡新建改建道路，综合配套交通标识、绿化亮化和地下管网，综合配套道路片区的学校、医院、商场等公益设施。三是改旧城，建设安居环境。启动洞庭风光带三期和洞庭新城建设，实施一批旧城改造项目。四是促转型，推进“退二进三”。抓好际华3517、市氮肥厂、市水泥厂、同联药业、新泰化肥、中南科伦等企业“退二进三”，优化产业布局。五是优环境，加强水系保护和治理。完成环南湖截污管网工程，抓紧做好内湖、内河综合配套治理前期工作。园区建设升温。大力推进项目满园扩园行动，吸引更多同类企业、上下游企业、关联企业和服务性企业入园集聚。大力推进园区产业升级行动，支持园区企业技术改造、做大做强，引进一批战略投资者落户。大力推进园区服务配套体系建设，不断优化发展环境。大力推进园区好项目竞赛活动，主要考核投资强度、项目收益、科技含量、节能环保，提高园区产业竞争力，着力打造千亿基地、百亿园区。港区建设加速。一是抓物流，形成车水马龙新气象。拓展城陵矶港经济腹地，组织更多货源进出，力争集装箱达到18万标箱以上。二是建平台，创造方便快捷新优势。完成“三纵三横”主干道路建设，推进公路、铁路向港区延伸，抓好水系治理；完善电子信息平台建设，开辟绿色通关通道。三是引产业，打造以港兴业新特色。围绕现代装备制造业、高新技术产业、综合物流业和配套服务业，招大引强，力争5大规划区都有重大项目布局，确保签约项目30个以上，入园项目20个以上。景区建设升级。推动各类景区提档升级，创建更多A级景区，确保“楼岛湖”进入5A级景区。加快屈子文化园、洞庭湖国际公馆、顺天洋沙湖度假村、盘石洲度假区、君山野生荷花世界二期工程等景区建设，完成洞庭山庄升级改造，提升张谷英景区品位。加快宾馆、市游客信息咨询服务中心等配套设施建设。农业区建设见效。加快屈原国家级现代农业示范区、湘阴全国农业标准化示范县、君山全国农产品加工示范基地、岳阳县台湾农民创业园、汨罗和华容两个省级农业标准化示范县建设，壮大平江油茶、临湘楠竹等特色产业，引领现代农业发展，促进农民增收。加大以平江为重点的扶贫开发力度。发展农业主导产业，整治村容村貌，推进新农村建设。

（二）力解“五题”。项目支撑：重点推进210个重大项目建设。一是推进炼化一体化、华能岳阳电厂三期、福坤汽车、远大科技、凯达科旺、富邦瑞博等50个重大产业项目建设。二是推进118个重大基础设施项目建设。加快6条高速公路、临湘至湖滨公路、芙蓉大道北拓至湘阴工程、炼化一体化公路、鲇鱼须大桥建设；S202二期、S306华容段、荣鹿公路等建成通车；力争开工建设S306南江桥至岳阳公路、S207平江段、许墨核电专用公路；完成农村公路建设600公里；启动漕溪港码头二期、鸭栏码头、登旺汽车站等建设。完成38座病险小（一）型水库除险加固；推进钱粮湖等蓄洪安全工程建设；实施许家台、中洲等大型灌排泵站更新改造；加快11条中小河流治理。三是推进临湘城镇污水处理、松阳湖治污、湘江流域重金属污染治理等26个生态项目建设。四是解决30万农村人口饮水安全问题，推进农村沼气工程、城乡电网改造工程等16个重大民生保障项目建设。资源要素：加快项目建设，关键是要确保生产要素供给。一是强化资金保障。发挥金融主渠道作用，改善金融生态，抓好招商引行，新增1家股份制商业银行、1家农村商业银行；深化银企合作，新增贷款100亿元以上；完善投融资平台，以项目公司运作模式加大融资力度，防范风险；支持优势企业上市融资、上市企业再融资，新增1家企业上市，2至3家企业在天津股权交易所挂牌；发展村镇银行和投资担保公司，激活民间投资；加大向上争取项目和资金的力度，确保我市争资份额占全省总额的十分之一以上。二是强化用地保障。在保护好耕地的基础上千方百计化解用地难题，大力推进土地的节约集约利用；加大清收闲置用地力度；推行建设用地增减挂钩；积极向上争取用地指标，最大限度满足项目用地需求。三是保护和利用好矿产资源。发展临湘等地矿产加工业。规范矿产资源开发秩序，加大矿山地质环境保护和恢复治理力度。发展环境：一是积极推进电子政务建设，规范收费项目，推广一票制收费，健全土地网上招拍挂。二是所有乡镇建立便民服务中心，半数以上的村建立代办服务点。三是严格依法行政，切实规范行政裁量权。四是认真开展政风行风评议，健全优化经济环境测评制度，以行政监察促进行政效能提速。五是强化行政问责，严肃查处扰乱经济发展环境的人和事。以优良的发展环境，扩大招商引资，力争全年到位内资160亿元、外资1.65亿美元。继续开展联手帮扶企业和产业发展升级行动。节能减排：我市产业结构决定了节能减排压力巨大，必须下决心抓实抓好。一是抓年耗能1万吨标准煤以上企业节能工作，加快淘汰落后产能，新上一批节能技术改造项目。二是抓农业面源污染治理，提高水环境质量。三是巩固提高湘江流域和环洞庭湖小企业污染整治成果。四是建成黄梅港等污水处理厂。五是抓产业转型升级，努力实现“两型”发展。安全稳定：坚决防范遏制重特大安全事故，严厉打击黑恶势力和刑事暴力犯罪，着力化解信访热点难点问题，抓好流动人口和特殊人群管理。积极稳妥推进各项改革，加快市属国有企业改制扫尾。

（三）深化“五创”。城乡、机关和企业联动，在全市范围整体推进“五创提质”。突出“十大重点”：一是实施“市民文明素质提升”行动，开展争做“文明岳阳人”活动，宣传普及“市民文明公约”。二是大力实施节能减排、流域治理和城乡环境卫生整治行动，加快推进垃圾处理“三年行动”计划。三是整治交通秩序，治理交通拥堵，缓解停车难。四是实施部分主次道路“白改黑”和重要节点部位亮化、美化、绿化；实现主次街道24小时、小街小巷16小时保洁。五是抓好殡葬改革和城区“禁炮”。六是大规模改造农贸市场，大力整治“五小”门店，强化食品药品安全监管。迎接国家卫生城市复查。七是坚决制止乱搭乱建乱挂，整治违法建筑。八是加强社会治安综合治理，扫黑除恶。九是改造中心城区小街小巷，建设一批市民小游园和过街设施。十是推行标准化、数字化、网格化城管，提升城市管理水平。

（四）实现“五有”。就业有增长。实施更加积极的就业政策，切实抓好就业与再就业，新增城镇就业4.2万人、农村劳动力转移就业7万人、失业人员再就业2.7万人，零就业家庭动态清零。收入有提高。努力提高城乡居民收入，最低工资标准提高25%，城市低保月标准提高到300元，农村低保月标准提高到135元。新型农村社会养老保险覆盖面50%以上。大力推行工资协商制。落实教师绩效工资。住房有改善。不断完善住房保障，推进房地产业健康发展；新建筹建廉租住房2000套、公共租赁住房1000套；新增经济适用房货币补贴1000万元以上；建成经济适用房12万平方米、中心城区集体土地拆迁安置房20万平方米；完成棚改1000户。教育有发展。坚持优先发展教育的方针不动摇，切实抓好国家教育规划纲要、湖南省建设教育强省规划纲要的落实，努力提高教育质量。完成校舍安全工程建设60万平方米，优化教育布局，努力化解教育负债。建设市青少年体育活动中心和市十五中。启动金凤桥片区综合性学校的前期工作。医疗有保障。继续抓好特困家庭大病医疗慈善救助工作。国家基本药物制度覆盖面达到66%，新型农村合作医疗参合率巩固在95%以上，完成3所县级综合医院建设。与此同时，坚持为民办实事，关注困难群体，切实做到发展为了人民、发展依靠人民、发展成果由人民共享。

各位代表，未来5年的蓝图已经绘就，新一年的工作已经铺开。我们坚信，任何困难都改变不了岳阳人民创造幸福美好生活的强烈愿望，任何挑战都阻挡不了我们建设“五市一极”的前进步伐！让我们在中共岳阳市委的坚强领导下，凝聚全市人民的智慧和力量，再接再厉，锐意进取，为实现岳阳经济转型升级、更大更强，为全面建成小康社会而努力奋斗！
